# Upregulation of cyclooxygenase-2 is accompanied by increased expression of nuclear factor-*κ*B and I*κ*B kinase-*α* in human colorectal cancer epithelial cells

**DOI:** 10.1038/sj.bjc.6600927

**Published:** 2003-05-13

**Authors:** M P Charalambous, C Maihöfner, U Bhambra, T Lightfoot, N J Gooderham, The Colorectal Cancer Study Group

**Affiliations:** 1 Molecular Toxicology, Division of Biomedical Sciences, Faculty of Medicine, Imperial College of Science, Technology and Medicine, London SW7 2AZ, UK; 2 Department of Neurology, University of Erlangen-Nürnberg, Schwabachanlage 6, 91054 Erlangen, Germany; 3 JBUEC, Department of Biology, University of York, York, Y01 5DD, UK

**Keywords:** colorectal cancer, immunohistochemistry, cyclooxygenase-2, nuclear factor-*κ*B, I*κ*B kinase-*α*

## Abstract

Cyclooxygenase-2 (COX-2) is selectively overexpressed in colorectal tumours. The mechanism of COX-2 induction is not fully understood, but requires *de novo* messenger RNA and protein synthesis, indicating regulation at the transcriptional level. Sequence analysis of the 5′-flanking region of the COX-2 gene shows two nuclear factor-*κ*B (NF-*κ*B) sites. Inhibition of this protein in model cell culture systems attenuates COX-2 expression and implies that NF-*κ*B plays an important role in COX-2 induction. We measured COX-2, NF-*κ*B and I*κ*B kinase *α* (IKK*α*) protein expression in matched colonic biopsy samples comprising both nontumour and adjacent tumour tissue from 32 colorectal cancer patients using immunohistochemistry. There was none or very little expression of COX-2, NF-*κ*B and IKK*α* in non-neoplastic colon epithelial cells, while the expression of all three of these proteins was significantly increased (*P*<0.05, Wilcoxon's signed rank test) in adjacent cancerous cells. Moreover, all three proteins were found to be coexpressed in the neoplastic epithelium, with the expression of COX-2 and NF-*κ*B highly correlated (Pearson's correlation, *P*<0.005). There was no apparent correlation between enhanced COX-2, NF-*κ*B or IKK*α* expression and tumour Dukes' stages. Our results are compatible with the hypothesis that IKK*α* and NF-*κ*B are involved in COX-2 induction in these tumours and the lack of association between COX-2 expression and severity of disease as measured by Dukes' stage is consistent with the proposal that COX-2 expression is an early postinitiation event.

Cyclooxygenases (COX) are intracellular proteins that catalyse the rate-limiting step in the synthesis of prostaglandins (PGs), a potent group of autocrine and paracrine lipid mediators (Mitchell *et al*, 1995; Smith *et al*, 1996; Chen *et al*, 2000) that are implicated in many normal cellular and pathophysiological processes (Mitchell *et al*, 1995; Portanova *et al*, 1996; Smith *et al*, 1996; Chen *et al*, 2000). Two forms of COX have been identified to date: the constitutively expressed form COX-1, and the inducible form COX-2. The role of COX-2 has been previously studied in colorectal cancer, and shown to be expressed in adenomas and carcinomas (Eberhart *et al*, 1994; Kargman *et al*, 1995; Sano *et al*, 1995; Dimberg *et al*, 1999; Cianchi *et al*, 2001; Denkert *et al*, 2001). COX-2 is believed to play an important role in colon carcinogenesis and several mechanisms could account for the link between the activity of COX-2 and carcinogenesis, including activation of procarcinogens (Wiese *et al*, 2001); production of PGs that can promote angiogenesis (Tsujji *et al*, 1998), inhibit immune surveillance and increase cell proliferation (Sheng *et al*, 1998); and direct inhibition of cell apoptosis (Tsujji and DuBois, 1997).

The mechanism of COX-2 induction in colon cancer is not fully understood, although it is known that induction requires messenger RNA and protein synthesis (Hempel *et al*, 1994), indicating regulation at the transcriptional level. The COX-2 gene can be induced by a wide variety of stimuli including oncogenic viruses, growth factors, cytokines and tumour promoters. Overexpression of COX-2 caused by hypoxia in human umbilical vein endothelial cells and in human alveolar epithelial cells, and by interleukin-1 (IL-1) in rheumatoid synoviocytes has been shown to be mediated by the nuclear factor-*κ*B (NF-*κ*B). Recently, inhibition of this latter pathway *in vitro* by curcumin (an inhibitor of NF-*κ*B activation) has been shown to attenuate COX-2 expression in colon cells (Plummer *et al*, 1999), indicating that NF-*κ*B may play an important role in COX-2 induction. Should this be the case in neoplastic cells, then the NF-*κ*B control of COX-2 expression would be important in the development and progression of human colorectal carcinoma.

We have therefore examined tissue biopsies obtained from patients with diagnosed primary colorectal cancer undergoing surgical treatment for their disease, for differences in the expression of COX-2 in malignant and adjacent normal epithelial cells, and for alterations in the expression of the upstream intracellular proteins that appear to be linked to COX-2 expression, specifically NF-*κ*B and I*κ*B kinase *α* (IKK*α*).

## MATERIALS AND METHODS

### Patients

Surgical specimens of primary tumours were obtained with informed consent from 32 patients with histologically verified colorectal cancer, treated at the Department of Surgery, York District Hospital, York, UK. Ethical approval for the study was obtained from the Human Research Ethics Committee at York District Hospital. Tumours were classified according to the Dukes' classification (see Table 1

**Table 1:** Patient demographic information

**Patient code**	**Age**^a^	**Sex**	**Tumour site**	**Dukes' stage**	**Drug history**	**Tobacco use**	**Alcohol**^b^
3053	72	M	Colon	B	None	No	8+
3501	68	M	Colon	C	NA	No	NA
3506	66	F	Colon	C1	5-Fluorouracil, Enalapril	No	0
3507	69	M	Rectum	C	Co-codamol	No	1–7
3509	49	F	Colon	B	NA	Yes	NA
3510	63	F	Rectum	NA	NA	No	NA
3512	52	M	Rectum	C1	Adalat	No	1–7
3515	75	M	Colon	C	NA	No	NA
3516	68	M	Colon	B	Atenolol, Prednisolone, Warfarin, Diltiazem, Isosorbide, Gliclazide, Co-danthramer	No	1–7
3517	69	M	Rectum	C1	Atenolol	No	8+
3518	70	F	Rectum	B	Lithium, Thyroxine	No	8+
3519	72	M	Rectum	B	Captopril, Naproxen, Allopurinol, Isosorbide, Frusemide, Atenolol, Prochlorperazine	No	1–7
3521	78	M	Colon	A	NA	No	NA
3522	56	M	Colon	A	None	No	8+
3524	76	M	Colon	A	None	No	1–7
3525	44	M	Colon	C	NA	No	NA
3528	58	F	Colon	NA	None	No	0
3529	61	F	Rectum	A	NA	No	NA
3531	66	F	Colon	B	None	No	0
3532	54	M	Rectum	C1	None	No	1–7
3533	49	M	Colon	B	None	No	8+
3534	73	F	Colon	B	NA	Yes	NA
3535	52	M	Rectum	B	None	No	8+
3536	68	M	Colon	B	Salbutamol, Ferrous sulphate	No	0
3537	63	F	Rectum	A	Salbutamol, Beclomethasone, Bendrofluazide	No	1–7
3538	56	M	Colon	C	Losec	No	8+
3540	68	M	Rectum	B	Sotalol, Aspirin	No	8+
3542	80	F	Rectum	B	None	No	0
3548	59	M	Rectum	C1	None	No	8+
3550	66	M	Rectum	NA	None	Yes	8+

NA=Not available.

a Age in years.

b Alcohol consumption in units per week (1 unit=half a pint of beer or one glass of wine or one shot of spirits).

). The entire study was carried out blind using coded tissue sections.

### Tissue specimens

Tissue samples taken at operation for histopathological confirmation of disease were fixed in 4% buffered formaldehyde and embedded in paraffin wax; sections surplus to pathology requirements were made available for this study. For 24 patients, tissue sections of both normal and malignant colon or rectum were provided (matched samples), while for the other eight patients only sections of malignant tissue were available. Only matched tissue was used for statistical analysis.

### Antibodies

The goat and rabbit polyclonal immunoglobulins (IgGs) for anti-human COX-2 and NF-*κ*B-p65 were as previously described (Maihöfner *et al*, 2000). The rabbit polyclonal IgG for anti-human IKK*α* was obtained from Santa Cruz Biotechnology Inc. (Santa Cruz, USA).

### Immunohistochemistry

The expression of COX-2, NF-*κ*B and IKK*α* in normal and malignant human colon epithelial cells was determined using a modified avidin/biotin immunohistochemistry procedure (Goggi *et al*, 1986). In preliminary experiments, each of the immunohisto-chemistry assays was optimised using a range of antisera dilutions (1/200–1/5000). For each assay, the negative control antisera (preimmune sera) were confirmed negative for staining at the dilution optimised for the primary antibody and blocking peptides (Santa Cruz Biotechnology Inc.) confirmed specificity. The dilutions used were 1/1500, 1/1000 and 1/800 for the anti-COX-2, NF-*κ*B and IKK*α* antibodies, respectively. The sections were deparaffinised and rehydrated through xylene and a series of graded alcohol solutions. Endogenous peroxidase activity was blocked by immersing the sections into a solution of 3% hydrogen peroxide in distilled water for 30 min at room temperature, and then rinsed in cold running tap water for 10 min. Incubating the sections with 5% normal swine serum for 30 min at room temperature reduced nonspecific background staining. Sections were then washed twice with phosphate-buffered saline (PBS) (5 min per wash) and 1 ml of either the primary antibody or the normal goat or rabbit IgGs (negative control) was applied to each section, and left at 4°C overnight. The next day, the slides were washed twice with PBS (5 min per wash), and then incubated with the secondary antibody solution (Biotinylated Swine anti-goat, mouse, rabbit immunoglobulin; 1/150 dilution; 1 ml per section) for 1 h at room temperature. After being washed twice with PBS (5 min per wash), they were incubated with the StreptABComplex solution (1 ml per section) for 1 h at room temperature, washed twice with PBS (5 min per wash) and immersed into the substrate (300 ml PBS, 90 *μ*l hydrogen peroxide and 2.5 ml 3,3-diaminobenzidine) for 3 min, and then rinsed with PBS and cold running tap water (5 min each). Sections were then successively immersed into haematoxylin, acid alcohol and Scott's tap water to counterstain. Finally, the sections were dehydrated by successive immersion into 70% ethanol, 100% ethanol twice and xylene twice and mounted.

### Immunohistochemical evaluation

**Figure 1:**
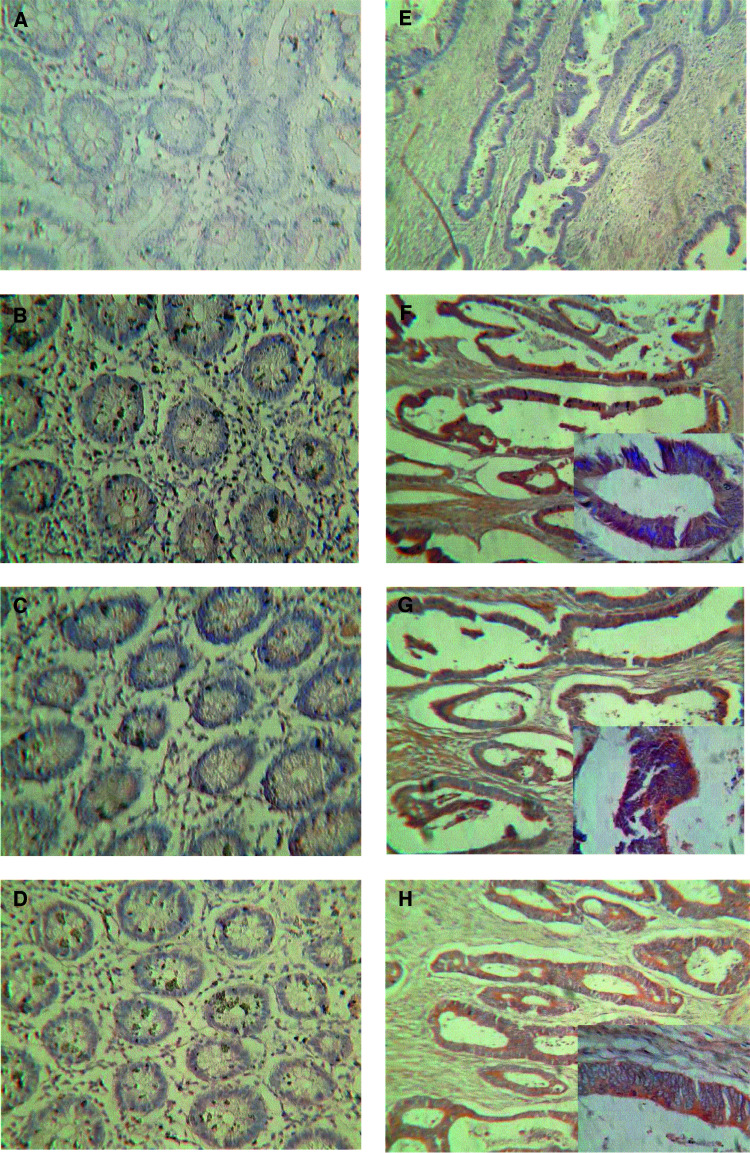
Immunohistochemical localisation of COX-2, NF-*κ*B and IKK*α* in normal and malignant colonic epithelia from the same patient. The presence of the immunoreactive protein is indicated by brown staining. (**A**) Normal and (**E**) tumour tissue treated with preimmune sera as primary antibody (negative control); (**B**) normal and (**F**) tumour tissue treated with anti-COX-2 as primary antibody; (**C**) normal and (**G**) tumour tissue treated with anti-NF-*κ*B primary antibody; (**D**) normal and (**H**) tumour tissue treated with anti-IKK*α* primary antibody. Insets are higher magnification of the same section.

Processed specimens were scored under the light microscope and the extent and intensity of staining with COX-2, NF-*κ*B and IKK*α* antibodies graded blind using coded slides. In order to assess and grade intensity and distribution of immunoreactivity in the colonic epithelium, a scoring method that has been previously described (Yukawa *et al*, 1994) was used. The distribution was scored according to the number of positive cells: none (not stained), 0; focal (<1/3 of cells stained), 1; multifocal (1/3–2/3 of cells stained), 2; and diffuse (>2/3 stained), 3. The staining intensity was scored as: none (not stained), 0; mild (between 0 and 2), 1; and strong, 2. The distribution and intensity scored were added to produce the following grades for the staining: 0, negative; 2, intermediate; and 3, 4 and 5, positive. Sections treated with the normal goat or rabbit IgGs (negative controls) or omitting the primary antibody were devoid of staining (Figure 1

). Positive staining controls for COX-2 included sections of brain, kidney and uterus. Representative examples of staining for COX-2 are shown in Figure 1.

### Statistical analysis

The Wilcoxon's signed rank test was used to compare the scoring of the respective immunoreactivity for COX-2, NF-*κ*B and IKK*α* between malignant and control epithelial tissues. The Pearson correlation test was used to assess the relation between COX-2 expression and NF-*κ*B and IKK*α*, and additionally to assess correlation between COX-2, NF-*κ*B and IKK*α* and Dukes' stages.

## RESULTS

### Expression of COX-2 in normal and malignant colorectal epithelial cells

**Figure 2:**
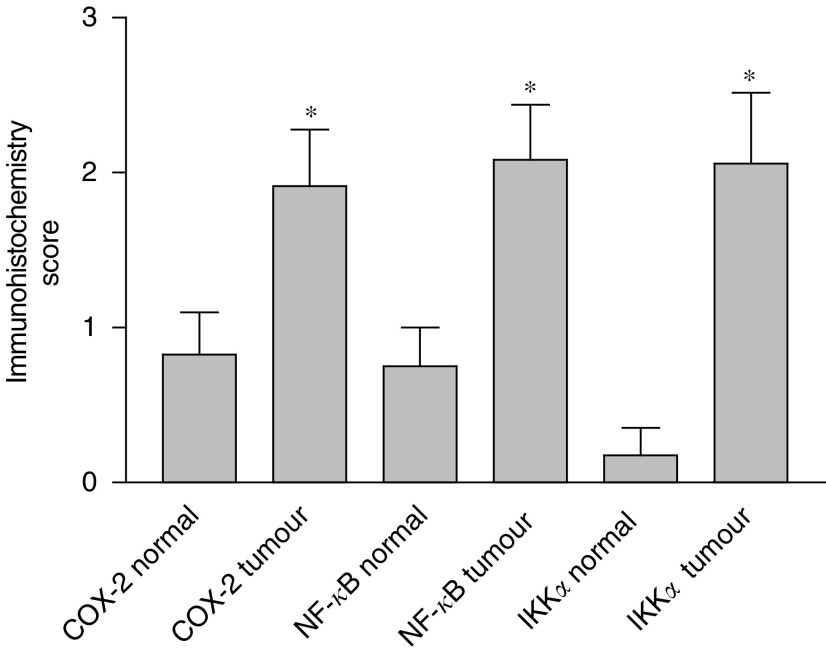
Expression of COX-2, NF-*κ*B and IKK*α* in matched normal and malignant colonic epithelia from 24 patients. ^*^Significantly different (Wilcoxon's signed rank test, *P*<0.03) from normal tissue.

Tissue sections of normal and malignant large bowel from colorectal cancer patients were investigated for COX-2 expression by immunohistochemistry. There was little cytoplasmic expression of COX-2 in non-neoplastic colonic and rectal epithelial cells (five out of 23 patients, mean rating score 0.826), consistent with the fact that COX-2 is an inducible enzyme. Yet in both colonic and rectal malignant epithelial cells, there was good COX-2 expression (17 out of 30 patients, mean rating score 1.913) (Figure 1). The staining was cytoplasmic and particularly concentrated around the nucleus, which is consistent with the known localisation of COX (rough endoplasmic reticulum and inner nuclear membrane). No staining was observed inside the nuclei of the epithelial cells. In those non-neoplastic tissue samples in which immunoreactive staining for COX-2 was detected, a similar pattern of expression was observed. Moderately and well-differentiated neoplastic epithelial cells showed significantly higher immunoreactivity than poorly differentiated tissues. Statistical analysis was applied to matched (nonmalignant *vs* malignant tissue from the same patient) samples (see Figure 2

), and demonstrated a significantly higher rating of the respective intensity scores for colorectal cancer epithelium compared to control cells (Wilcoxon's signed rank test; *P*<0.027 for COX-2).

### Expression of NF-*κ*B-p65 in normal and malignant colorectal epithelial cells

Tissue sections of normal and malignant large bowel from colorectal cancer patients were also investigated for NF-*κ*B-p65 expression. The majority of the non-neoplastic colon and rectum specimens showed either none at all or very little cytoplasmic expression of NF-*κ*B-p65 (four out of 24 patients, mean rating score=0.750), and no staining was observed inside the nuclei (Figure 1). In contrast, in more than half of the colonic and rectal malignant epithelial cells there was an observable increase of NF-*κ*B-p65 expression, which was mainly cytoplasmic but with some staining observed inside the nuclei (17 out of 32 patients, mean rating score=2.083). Moderately and well-differentiated malignant epithelial cells showed significantly higher immunoreactivity than poorly differentiated cells. For the matched samples, the majority of patients had a measurable increase in the expression of immunoreactive NF-*κ*B-p65 in malignant cells compared to their matched nonmalignant tissue (13 out of 24 patients). Statistical analysis (Wilcoxon's signed rank test) of the matched patient samples (Figure 2) showed that there was a highly significant increase in the mean expression of NF-*κ*B-p65 between normal and malignant colorectal epithelial cells (*P*<0.027).

### Expression of IKK*α* in normal and malignant colorectal epithelial cells

There was little expression of immunoreactive IKK*α* in non-neoplastic colonic or rectal epithelial cells (1 out of 17 patients, mean rating score=0.176), indicating that IKK*α* is not constitutively expressed in significant amounts in these cells. Nevertheless, in both colonic and rectal malignant epithelial cells, there was an increase of IKK*α* expression compared to non-neoplastic tissue (12 out of 24 patients, mean rating score=2.059) (Figure 1). Examination of the matched samples for changes in the expression of IKK*α* showed that the majority (10 out of 17 patients) had an increase in expression in the malignant compared to the nonmalignant cells. This was statistically significant (*P*<0.027) for matched tissue pairs (Figure 2). The IKK*α* expression was mainly cytoplasmic, and no staining was observed inside the nuclei of the epithelial cells. There was a significantly higher immuno-reactivity of the protein in moderately and well-differentiated cancerous epithelial cells than in the poorly differentiated cases.

### Coexpression of COX-2, NF-*κ*B and IKK*α* in malignant colorectal epithelial cells

**Figure 3:**
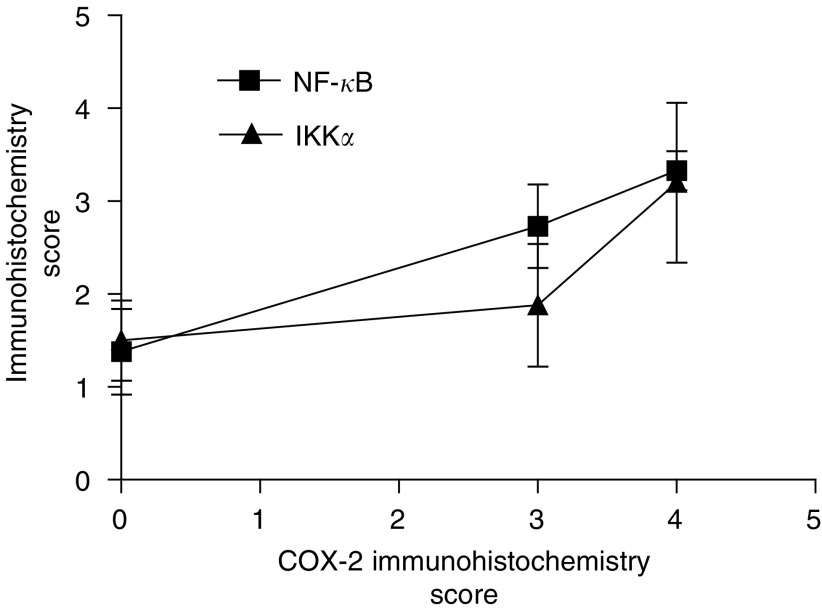
Expression of NF-*κ*B and IKK*α* compared to COX-2 in malignant colonic epithelia. Expression of NF-*κ*B is highly correlated with COX-2 expression (Pearson's correlation, two-tailed *P*-value <0.005, *n*=30 patients). Values are mean±s.e.m.

In order to determine histologically if there was coexpression of protein in malignant tissue, serial tissue sections were examined for expression of COX-2, NF-*κ*B and IKK*α*. In by far the majority of cases (15 out of 17), a positive level of COX-2 immunoreactivity in neoplastic tissue was accompanied by positive levels of NF-*κ*B and/ or IKK*α* immunoreactivity (Figure 1) supporting the proposal that the three proteins were coexpressed. This was particularly evident in moderately to well-differentiated tissues. In agreement with this histological finding, there was a highly significant correlation between COX-2 and NF-*κ*B expression in malignant epithelium (Pearson's correlation test, two-tailed, *P*<0.005, *n*=30) (Figure 3

). In the case of IKK*α* immunoreactivity, although mean expression increased with mean COX-2 expression, a linear correlation was not evident (Figure 3).

### Association between COX-2, NF-*κ*B or IKK*α* and severity of colorectal cancer

Comparison of the expression of COX-2, NF-*κ*B or IKK*α* and Dukes' stage showed no significant association (Pearson's correlation test).

## DISCUSSION

We found little expression of COX-2 in non-neoplastic colorectal epithelial cells, reflecting the fact that COX-2 is an inducible enzyme with low basal expression. However, in both colonic and rectal malignant epithelial cells, there was a trend for increased COX-2 expression, which is consistent with previous reports (Eberhart *et al*, 1994; Kargman *et al*, 1995; Sano *et al*, 1995; Dimberg *et al*, 1999; Shattuck-Brandt *et al*, 1999; Cianchi *et al*, 2001; Denkert *et al*, 2001). A similar trend was observed for both colonic and rectal malignant epithelial cells, with less than half of the patients showing no expression of COX-2 (13 out of 30 patients), about a third showing some immunoreactive staining (score 2 or 3) and the rest showing significant expression (score 4 or 5) (6 out of 30 patients). These results suggest that a similar mechanism of COX-2 induction is involved in both rectal and colonic malignant epithelial cells. This is in contrast to a recent Swedish study (Dimberg *et al*, 1999), in which there was a notable overexpression of COX-2 protein in tumours located in the rectum, compared with other locations in the colon, as measured by Western blotting. However, many other studies have investigated COX-2 expression in colorectal cancer, and none has reported a discrepancy in COX-2 expression between colon and rectal malignant epithelial cells.

NF-*κ*B is an inducible higher eukaryotic transcription factor, which has a pivotal role in the regulation of the expression of many genes involved in immune and inflammatory responses (Sha, 1998; Bowie and O'Neill, 2000), the replication and reactivation of many viruses, in neuronal development and neurodegeneration, and in the control of cell proliferation and apoptosis (Baeuerle and Henkel, 1994; Pahl and Baeuerle, 1995; Baeuerle and Baltimore, 1996; Bowie and O'Neill, 2000). NF-*κ*B can be activated in response to a broad range of stimuli and conditions, including bacterial and viral products, inflammatory cytokines such as IL-1 and tumour necrosis factor (TNF), B- and T-cell mitogens, intracellular stresses such as endoplasmic reticulum overload, and extracellular stresses like asbestos fibres, ultraviolet light and cigarette smoke (Baeuerle and Henkel, 1994; Janssen *et al*, 1995; Pahl and Baeuerle, 1995; Shen *et al*, 1996; O'Neil and Kaltschmidt, 1997; Bowie and O'Neill, 2000). NF-*κ*B is present in the cytoplasm of unstimulated cells in a latent form, comprising a transcriptionally active dimer bound to an inhibitory protein, I*κ*B (Sha, 1998). Both NF-*κ*B and I*κ*B exist in multiple forms (Baeuerle and Baltimore, 1996).

The mechanism by which diverse stimulants activate NF-*κ*B involves the phosphorylation of I*κ*B*α* on two serine residues (S32 and S36). This causes the release of the NF-*κ*B dimer, which can then translocate to the nucleus and activate target genes by binding with high affinity to *κ*B elements in promoters (Bowie and O'Neill, 2000). To date, two I*κ*B kinases have been identified, termed IKK*α* and IKK*β* (Whiteside and Israel, 1997). These two proteins have been shown to be activated by inducers of NF-*κ*B, such as IL-1 and TNF, to phosphorylate S32 and S36 of I*κ*B, and to play a key role in the activation of NF-*κ*B by these cytokines (Mercurio *et al*, 1997; Stancovski and Baltimore, 1997; Bowie and O'Neill, 2000). The IKKs are part of a larger multiprotein complex called the IKK signalsome, which contains the IKK complex-associated protein (IKAP) and IKK*γ* (also called NEMO), which is also crucial for NF-*κ*B activation (Cohen *et al*, 1998; Yamaoka *et al*, 1998; Bowie and O'Neill, 2000). Many upstream activators and regulators of IKK activity have been identified, including the NF-*κ*B-inducing kinase (NIK) (Nakano *et al*, 1998), protein kinase C*ζ* (PKC*ζ*) (Lallena *et al*, 1999), transforming growth factor *β*-activated kinase (TAK-1) (Ninomiya-Tsuji *et al*, 1999), MEK kinase (MEKK) 1, 2 and 3 (Zhao and Lee, 1999), and S6 kinase (Schouten *et al*, 1997). NF-*κ*B can induce the expression of numerous target genes, including COX-2. In model systems, overexpression of the COX-2 protein caused by hypoxia in human umbilical vein endothelial cells and by IL-1 in rheumatoid synoviocytes has been shown to be mediated by NF-*κ*B (Schmedtje *et al*, 1997; Chen *et al*, 2000). Furthermore, there are two NF-*κ*B consensus sites present in the promoter region of the human COX-2 gene (Tazawa *et al*, 1994), these being the NF-*κ*B-3′ site (−223/−214) and the NF-*κ*B-5′ site (−447/−438), indicating that NF-*κ*B may be involved in COX-2 induction (Bowie and O'Neill, 2000).

From the foregoing, there is evidence from model systems that COX-2 induction involves NF-*κ*B. Our investigations show that cytoplasmic NF-*κ*B-p65 protein (which is thought to be mostly in its inactive, bound state) is present in small amounts in non-neoplastic colorectal epithelial cells, while no endonuclear (unbound, active form) protein was detected in these cells. In contrast, in malignant epithelial cells of the colon and rectum, there was a distinct increase of both inactive (cytoplasmic) and potentially active (endonuclear) NF-*κ*B-p65. Thus in malignant colorectal epithelial cells, both the expression of the NF-*κ*B gene, as well as the activation of the latent cytoplasmic protein to an active form are increased. The intracellular, cytoplasmic concentration of IKK*α* also tended to be expressed at increased levels in cancerous colorectal epithelial cells. Furthermore, in tumour tissue the expression of NF-*κ*B-p65 was highly correlated with COX-2 immunohistochemistry. These observations are compatible with the hypothesis that NF-*κ*B is involved in COX-2 induction in these tumours, and that NF-*κ*B is probably activated by IKK*α*.

This is in agreement with many epidemiological studies, which have demonstrated a 40–50% decrease in relative risk for colorectal cancer in individuals taking nonsteroidal-antiinflammatory drugs, compared to those not taking these agents. In addition, several lines of experimental evidence indicate that NF-*κ*B may play an important role in the development and/or progression of human cancers: (1) several members of the I*κ*B and NF-*κ*B families derive from genes that are translocated or amplified in human cancers (Bours *et al*, 2000). These genetic changes can all lead to enhanced NF-*κ*B transcription activity, indicating that NF-*κ*B target genes may be involved in the control of crucial steps for cellular transformation and cancer progression (Bours *et al*, 2000). (2) The v-*rel* oncogene of the reticuloendotheliosis virus T (Rev-T), the first member of the Rel/NF-*κ*B family to be discovered (Gilmore, 1999; Bours *et al*, 2000), can directly transform cells *in vivo* or *in vitro*. (3) More recently, NF-*κ*B constitutive activity, as observed in Hodgkin's lymphoma cells, has been associated with a mutation in the gene encoding the I*κ*B-inhibitor (Krappmann *et al*, 1999), which can lead to impaired control of NF-*κ*B activity and hence to enhanced nuclear activity (Bours *et al*, 2000). The NF-*κ*B transcription factor is activated in response to a broad range of preapoptotic stimuli (Osborn *et al*, 1989; Brach *et al*, 1991; Schreck *et al*, 1991), dissociates from its attached inhibitory protein I*κ*B and translocates to the nucleus to induce the expression of target genes, including several well-known antiapoptotic genes such as TNF-receptor-associated factor 1 (TRAF1), and TRAF2, cIAPs, manganese superoxide dismutase, A20 and IEX-IL (Wang *et al*, 1998; Wu *et al*, 1998).

Although NF-*κ*B has been previously shown to be expressed at high levels in human colonic adenomatous polyps, our investigations have demonstrated for the first time (to the best of our knowledge) that IKK*α*, cytoplasmic inactive NF-*κ*B-p65 protein and putative active endonuclear NF-*κ*B-p65 protein are significantly increased in malignant colorectal epithelial cells. Our data support the findings of many *in vitro* experiments and provide evidence for a direct association between NF-*κ*B and COX-2 induction in human tumours.

In summary, we have shown that intracellular levels of NF-*κ*B-p65 and IKK*α* are increased in malignant colorectal epithelial cells, compatible with the hypothesis that NF-*κ*B is involved in COX-2 induction in these tumours, and possibly the activation of other antiapoptotic genes that influence the development of colorectal carcinogenesis. Finally, the lack of association between NF-*κ*B or COX-2 expression and Dukes' stages further suggests that NF-*κ*B and COX-2 expressions are possibly early postinitiation events that could be involved in tumour progression.

## Appendix 1

### The Colorectal Cancer Study Group

Dr J Barrett^1^, Professor DT Bishop^1^, Professor AR Boobis^2^, U Bhambra^2^, Professor D Forman^3^, Professor RC Garner^4^, Dr NJ Gooderham^2^, Dr TJ Lightfoot^4^, Dr C Sachse^5^, Dr G Smith^5^, Ms R Waxman^3^ & Professor CR Wolf^5^.

^1^Genetic Epidemiology, Cancer Research UK Clinical Centre, St. James's University Hospital, Leeds, UK, ^2^Faculty of Medicine, Imperial College London, UK, ^3^University of Leeds, UK, ^4^JBUEC, University of York, UK, ^5^Biomedical Research Centre, University of Dundee, UK.
